# Complete atrioventricular block during renal transplantation in a patient with Alport's syndrome: case report

**DOI:** 10.1590/S1516-31802001000500007

**Published:** 2001-09-01

**Authors:** Fábio Ferrari, Paulo do Nascimento, Pedro Thadeu Galvão Vianna

**Keywords:** Anesthesia, Renal transplantation, Complication: atrioventricular block, Anestesia, Transplante renal, Complicação: bloqueio atrioventricular total

## Abstract

**CONTEXT:**

Patients with Alport's syndrome (causing 5% of end-stage renal disease) have a higher risk of heart conduction abnormalities.

**OBJECTIVE:**

To report a case of Alport's syndrome developing complete atrioventricular block during renal transplantation.

**CASE REPORT:**

A 21-year-old man with chronic renal failure due to Alport's syndrome was submitted to a renal transplantation under epidural anesthesia and, during the intraoperative period, a complete atrioventricular block was diagnosed and promptly treated with a transcutaneous pacemaker. This extensive sympathetic block can contribute towards disturbances in the heart conduction system, particularly in patients with chronic renal disease in hemodialysis. Even in patients with a normal preoperative electrocardiogram or no conduction system disturbances, some degree of atrioventricular block, including complete atrioventricular block, can occur. In this situation, a transcutaneous pacemaker provides rapid and effective treatment in the operating room, thereby permitting the planning of a definitive treatment.

## RESUMO

**CONTEXTO:**

A síndrome de Alport é responsável por aproximadamente 5% dos pacientes com insuficiência renal crônica. Nessa doença, anormalidades no sistema de condução cardíaco são mais freqüentes que na população em geral.

**OBJETIVO:**

Relatar caso de síndrome de Alport que desenvolveu bloqueio atrioventricular total durante um transplante renal.

**RELATO DE CASO:**

Paciente masculino, 21 anos de idade, com insuficiência renal crônica secundária à síndrome de Alport, foi submetido a transplante renal sob anestesia peridural. Durante o procedimento anestésicocirúrgico apresentou bloqueio atrioventricular total, que foi tratado rapidamente, e com sucesso, usando-se um marcapasso trans-cutâneo. O bloqueio simpático extenso pode contribuir para o desenvolvimento de distúrbios no sistema de condução cardíaco, particularmente em pacientes com insuficiência renal crônica em esquema de diálise. Mesmo em pacientes com eletrocardiograma pré-operatório normal e sem distúrbios de condução, graus variáveis de bloqueio atrioventricular, incluindo bloqueio atrioventricular total, podem ocorrer. Nesse caso, o uso de marcapasso transcutâneo é tratamento rápido e efetivo na sala de operação até o final da cirurgia, quando o tratamento definitivo pode ser planejado.

## INTRODUCTION

Alport's syndrome is characterized by progressive nephritis that is more severe in males. It is manifested by hematuria and associated with a sensory nerve hearing deficit. The transmission is consistent with X-linked dominant inheritance and the syndrome accounts for nearly 5% of patients with end-stage renal disease. These patients can develop abnormalities in heart conduction due to hyperkalemia or the use of calcium channel inhibitors like verapamil and diltiazem.^1–4^ We report a case of complete atrioventricular block during renal transplantation surgery in a patient with chronic renal failure secondary to Alport's syndrome.

## CASE REPORT

A 21-year-old man with chronic renal failure secondary to Alport's syndrome, with no urinary output, and hypertension under treatment by captopril 25 mg per day, was called for urgent renal transplantation. He was submitted to hemodialysis before the surgery. His preoperative laboratory results were normal except for a serum creatinine concentration of 8.6 mg% and serum BUN concentration of 127 mg%. His hematocrit was 29% and his platelet count was 289,000/mm^3^. The preoperative electrocardiogram showed left ventricular hypertrophy.

In the operating room, arterial blood pressure was 180/110 mmHg, heart rate was 85 beats/min with sinus rhythm, and hemoglobin oxygen saturation was 98% without supplementary oxygen. Lumbar epidural anesthesia was performed at the L3-L4 space via a 17-gauge Tuohy needle in the sitting position, and 112.5 mg ropivacaine plus 2 mg morphine for postoperative analgesia were slowly injected. The sensory block reached a T4 level and stable hemodynamic conditions were observed (arterial blood pressure was 140/80 mmHg and heart rate was 75 beats/min).

**Figure 1:**
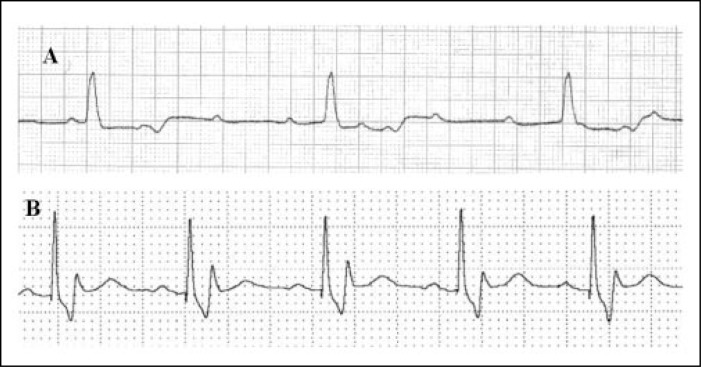
Electrocardiogram showing the complete atrioventricular block in the operating room (A) and with pacemaker rhythm in the Post Anesthesia Care Unit (B).

One hour after the beginning of the surgery, the patient's heart rate decreased to 30 beats/min and arterial blood pressure was 90/50 mmHg with an electrocardiograph trace showing complete atrioventricular block. Atropine 1 mg and calcium gluconate 1 g were injected intravenously with no response. The patient became unconscious and was immediately intubated. A transcutaneous pacemaker was introduced after about 5 minutes and heart rate was adjusted to 70 beats/min with pacemaker rhythm. A cannula was placed in the right radial artery for invasive blood pressure monitoring and medium arterial pressure was 80 mmHg, which then remained stable until the end of surgery. Serum potassium concentration was normal. We administered alfentanil 2.5 mg, atracurium 25 mg, and isoflurane to maintain anesthesia. Intraoperative course had no other problems. At the end of surgery a transvenous pacemaker was placed through the right internal jugular vein and the patient was extubated and transferred to the Post Anesthesia Care Unit (Figure 1) for recovery and hemodialysis, where no evidence for brain damage or myocardial infarction was found. The patient was scheduled for fitting with a definitive pacemaker.

## DISCUSSION

In this case report, the patient was young and the preoperative electrocardiogram displayed only left ventricular hypertrophy without conduction disturbances, and the serum potassium level was 4.8 mEq/l. Even so, he developed sudden complete atrioventricular block, which was successfully treated with the transcutaneous pacemaker during the intraoperative course.

The need for permanent pacing occurs more often in patients with chronic renal failure than it does in the general patient population. This could reflect an increase in conduction system disease caused by renal failure due to abnormalities in calcium metabolism leading to fibrosis and myocardial calcification.^5^

Calcium deposition in the cardiac conduction system induces progressive arrhythmia and one study has shown a high percentage of second- and third-degree atrioventricular block (69%) with dialysis.^6^ Among the factors postulated to promote the development of metastatic calcification, both elevated calcium-phosphorus product and increased parathyroid hormone levels are possible, and exploration or removal of the parathyroid glands should be considered if heart block is present.^7^

Another factor that influences cardiac conduction is serum potassium level. For levels above 7.0 mEq/l, intraventricular conduction velocity is usually decreased and the QRS complex widens. Although sinoatrial and atrioventricular nodes are apparently less sensitive to hyperkalemia than other cardiac fibers because of their calcium-dependent electrophysiological properties, atrioventricular block due to hyperkalemia can occur.^8,9^

Anesthetic technique may have been an important factor in the sudden cardiac block due to extensive sympathetic block. Epidural block with significant vasodilatation typically produces bradycardia and a depressed baroreceptor response, so that low pressures fail to induce accelerated heart rates. This change in baroreceptor performance does not require T1-T5 block, indicating that the heart rate response to blocks is not due to cardiac accelerator fiber block. Electrophysiological effects of segmental thoracic epidural block are similar to those of beta-adrenergic block, slowing conduction velocities and prolonging ventricular refractory periods. This may cause atrioventricular block.^10^

Occasionally, profound bradycardia and circulatory collapse may develop without any obvious precipitating event.^11^ While there may be many contributory factors, the trigger is probably low cardiac filling pressure causing intense reflex vagal activity and sympathetic withdrawal.^12^ At least three reflexes have been proposed. The first involves collapse-firing of low-pressure baroreceptors located in the right atrium and vena cava. A second reflex involves an arc located within the pacemaker cells of the myocardium, in which heart rate is proportional to the degree of stretch. Finally, there is a paradoxical Bezold-Jarisch response, in which mechanoreceptors located in the inferior posterior wall of the left ventricle can cause bradycardia when stimulated. In addition to these myocardial reflexes, a high level of sympathetic block may alter the balance of autonomic input to the heart, favoring vagal tone and bradycardia.^13^

Thus, in patients with chronic renal failure submitted to renal transplantation, we should probably avoid extensive sympathetic block due to its effects on the cardiovascular system and the potential additional effect on the cardiac conduction system, even when there is no evidence of dysfunction on the conduction system. These effects can lead to intense bradycardia and atrioventricular block, increasing the morbidity and compromising the renal transplantation. In this case, the transcutaneous pacemaker was a lifesaver because of its facility and rapidity in installation, even in the operating room where this complication is extremely rare, thereby permitting the planning of a definitive treatment.
